# New York Preparatory School of Medicine

**Published:** 1853-03

**Authors:** 


*New York Preparatory School of Medicine.*—The New York *Medical Times* contains an announcement of an association in the city of New York, under the above title. The design of the association is to "furnish to medical students the *elements* of a complete and scientific education, by means of *daily recitations* and familiar *demonstrations.*"

"The plan of instruction," it is stated, "is so arranged that the whole groundwork of practical medicine and surgery, with anatomy, physiology, and chemistry, may be advantageously gone over in one year, by means of *Systematic Text-books;* and at the same time a course of *specialties* will be followed with the aid of standard *monographs,* which will require three years for its completion."

The instructors are Prof. Ehrick Parmlee, M. D., Descriptive Anatomy; Prof. J. C. Dalton, M. D., Physiology and General Anatomy; J. Outram, jr., Chemistry and Materia Medica; A. K. Gardner, M. D., Midwifery and Diseases of Women; Henry Weeks Brown, M. D., Principles and Practice of Surgery; C. F. Heywood, M. D., General and Special Pathology, etc.

Most of the foregoing are new candidates for favor as medical teachers. Of one of the members of the school we may be permitted to speak from personal knowledge and association. The departments of physiology and general anatomy could hardly be intrusted to one better qualified to do them justice.

